# The effectiveness of psychological therapies for anxiety or depression, for people living with atypical dementia

**DOI:** 10.1002/alz.084145

**Published:** 2025-01-09

**Authors:** Celine El Baou, Rob Saunders, Joshua Buckman, Marcus Richards, Claudia Cooper, Georgia Bell, Caroline Fearn, Stephen Pilling, Sebastian J Crutch, Nikki Zimmermann, Emilie V Brotherhood, Amber John, Joshua Stott

**Affiliations:** ^1^ University College London, London United Kingdom; ^2^ Camden and Islington NHS Foundation Trust, London United Kingdom; ^3^ MRC Unit for Lifelong Health & Ageing at UCL, London United Kingdom; ^4^ Queen Mary University of London, London United Kingdom; ^5^ University College London, London, London United Kingdom; ^6^ Dementia Research Centre, UCL Queen Square Institute of Neurology, London United Kingdom; ^7^ Dementia Research Centre, UCL Queen Square Institute of Neurology, University College London, London United Kingdom; ^8^ Department of Clinical, Educational, and Health Psychology, Division of Psychology and Language Sciences, University College London, London United Kingdom

## Abstract

**Background:**

Prior research has highlighted that people living with dementia experiencing depression or anxiety can benefit from psychological therapies delivered in primary care. Yet, studies have mainly focused on common dementia types, leaving a gap in evidence for more atypical dementias, and especially those that are not memory‐led. This evaluation is crucial, given the unique challenges posed by these subtypes, such as syndrome‐specific symptoms of changes in personality or visual processing. This study assesses the effectiveness of psychological therapies provided in primary care settings for individuals with atypical dementia subtypes.

**Method:**

Linked national data from over 2 million individuals in the UK who received a course of therapy in NHS Talking Therapies for Anxiety and Depression services were used to identify 523 individuals with atypical dementias who attended therapy between 2012 and 2019. We compared their depression and anxiety outcomes to a cohort of individuals with no diagnosis of dementia.

**Result:**

People with atypical dementia had similar outcomes compared to people with typical dementia [Reliable improvement from depression and anxiety symptoms: AdjOR 1.08 (0.86; 1.36) p = 0.4850], but their therapy outcomes were poorer when compared to the matched cohort without dementia [Reliable improvement: AdjOR 0.70 (0.53; 0.91) p = 0.0150].

**Conclusion:**

People with atypical dementia who experience depression or anxiety may benefit from psychological therapies provided in primary care. Their outcomes are poorer than those of people who do not have dementia. These results suggest a need to increase access to psychological therapies for people with atypical dementia and offer interventions that are better tailored to their specific experiences.

